# Association between the serum uric acid to HDL cholesterol ratio and forearm bone mineral density in middle-aged and older adults

**DOI:** 10.3389/fendo.2026.1710027

**Published:** 2026-02-11

**Authors:** Fangping Song, Yao Sang, Xiuyan Fang, Zilong Tang, Ling Zhang

**Affiliations:** Department of General Practice, Heze Hospital Affiliated to Shandong First Medical University, Heze, Shandong, China

**Keywords:** uric acid/HDL cholesterol ratio, bone mineral density, subgroup analysis, interaction effect, forearm

## Abstract

**Background:**

Osteoporosis has emerged as a growing public health concern due to its high prevalence and substantial economic burden on both individuals and society. Recent studies have identified the serum uric acid to high-density lipoprotein cholesterol ratio (UHR) as a novel predictive biomarker for various diseases. However, its association with bone mineral density (BMD) remains unclear.

**Objective:**

This study evaluated the association of the UHR and forearm BMD (FR-BMD) in a middle-aged and elderly cohort. We also assessed the interaction effects of age, sex, and body mass index (BMI).

**Methods:**

A total of 4,958 adults aged ≥50 years were enrolled from health examinees at Heze Municipal Hospital (2022–2025). We collected demographic data, serum lipids, and uric acid levels. Measurements of FR-BMD were performed on the left distal radius (1/3 site) utilizing dual-energy X-ray absorptiometry. Multivariate linear regression analyses evaluated the UHR-BMD relationship, supplemented by subgroup analyses and interaction tests. Nonlinear associations were assessed using generalized additive models with smoothing curves.

**Results:**

After adjusting for age, sex, BMI, Alb, ALP, ALT, BUN, TP, Scr, Lp(a), TC, GGT and hypertension, a higher UHR was significantly associated with lower FR-BMD [β=-0.076, 95%CI(-0.138~-0.015), P = 0.015]. Significant interaction effects were observed for age and sex (*P for interaction* < 0.05). Subgroup analysis revealed that this inverse association was significant in males and overweight individuals (*P* < 0.05). In contrast, a positive association was observed in females aged ≥ 60 years [*β* = 0.278, *95%CI*(0.126~0.430), *P*<0.001]. Nonlinear analyses demonstrated a U-shaped relationship in those with BMI < 24 kg/m² (inflection point: 0. 102), an inverted U-shaped association in females ≥60 years (inflection point: 0. 156) and a significant but nonspecific nonlinear association was observed in males aged <60 years.

**Conclusion:**

The association of UHR with FR-BMD is significantly modified by age and sex in middle-aged and elderly populations. Nonlinear relationships exist in males <60 years, females ≥60 years and non-overweight individuals. The potential of UHR as a novel indicator for bone health assessment in select populations is highlighted by our results.

## Introduction

Osteoporosis (OP) is a chronic metabolic bone disorder characterized by diminished bone mass and microarchitectural deterioration of bone tissue, primarily resulting from an imbalance in bone remodeling due to suppressed osteoblastic activity and potentiated osteoclastic function (1). Bone mineral density (BMD) serves as a critical diagnostic indicator for osteoporosis. According to the 2018 national epidemiological survey on osteoporosis in China, the prevalence among individuals aged 50 years and older is 19.2% (32.1% in females and 6.9% in males) (1). This rate continues to rise. Osteoporosis and its complications, particularly osteoporotic fractures, present considerable risks to patient wellbeing while generating significant economic and psychological burdens for families and society (2). Healthcare costs in China attributable to osteoporotic fractures are projected to surge to 163 billion CNY by 2050 (3). The development of osteoporosis is closely linked to reduced BMD. Consequently, identifying additional risk factors associated with declining BMD is crucial for the early detection of osteoporosis and the prevention of its complications. The serum uric acid(SUA) to high-density lipoprotein cholesterol (HDL-C) ratio (UHR) has recently emerged as a novel indicator reflecting metabolic and inflammatory status (4), and has been associated with various cardiometabolic conditions (5–10). However, its relationship with BMD remains unclear, particularly the effect modifications by age and sex. No studies have systematically investigated the age-and sex-stratified associations between UHR and BMD or explored potential nonlinear trends. The primary objective of this study was to investigate how UHR correlates with BMD among middle-aged and older screening participants, focusing on the modifying effects of age and sex, and to assess the potential of UHR as an indicator for bone health assessment.

## Methods

### Study design and population

This investigation retrospectively analyzed data of 4,958 individuals who received systematic health evaluations at the Health Examination Center of Heze Municipal Hospital from January 2022 to May 2024. The inclusion criteria were: (i) aged ≥50 years; (ii) availability of complete data for all essential study variables (demographics, BMD, serum biomarkers). Individuals with any missing data were excluded, as the missingness was minimal (<0.5%) and assumed random. Exclusion criteria comprised: (i) history of malignancy, endocrine or metabolic disorders (including gout, hyperuricemia, thyroid diseases, or cushing syndrome), autoimmune diseases (rheumatoid disorders, systemic lupus erythematosus) or organ transplantation; (ii) chronic hepatic or renal diseases; (iii) acute inflammatory conditions. The research protocol obtained ethical clearance from the Medical Ethics Committee of Heze Municipal Hospital.

### BMD measurement and osteoporosis

Forearm bone mineral density (FR-BMD) was measured at the distal one-third radius of the left forearm using a dual-energy X-ray absorptiometry (DXA) scanner (model ALPHYS A, Fujifilm Medical Systems Suzhou Co. Ltd. China). BMD values are reported in grams per square centimeter (g/cm²). This site was selected because it is a non-weight-bearing bone, which may reflect systemic metabolic influences on bone density more directly than weight-bearing sites such as the hip or spine. Additionally, forearm DXA is widely used in epidemiological studies due to its convenience, lower radiation exposure, and applicability in large-scale health examinations. T-scores were automatically calculated by the manufacturer’s software. To ensure measurement precision, a strict quality control protocol was followed. Daily calibration was performed using the manufacturer-supplied phantom. Long-term instrumental stability was monitored through weekly phantom scans, which confirmed a maintained coefficient of variation (CV) of ≤1.5%. All scans were performed by certified radiologic technologists. BMD was classified into three categories based on the Chinese Clinical Guidelines for Primary Osteoporosis: normal (T-score≥-1.0), osteopenia (T-score between -2.5 and -1.0), and osteoporosis (T-score≤-2.5) (3). For this study, osteoporosis was identified by T-scores ≤ -2.5; all others were classified as non-osteoporosis.

### UHR assessment

The UHR was utilized as the primary explanatory variable, derived from the following equation: UHR = SUA (mg/dL)/HDL-C (mg/dL). Grouping was performed according to UHR quartiles (Q1-Q4) with the following ranges: Q1: UHR < 0.076, Q2: 0.076 ≤ UHR < 0.102, Q3: 0.102 ≤ UHR < 0.134, Q4: UHR ≥ 0.134.

### Study variables

Demographic data including age, sex, medical history, and surgical history were collected for all participants. The presence of comorbidities—specifically hypertension, diabetes mellitus, and cardiovascular disease (encompassing coronary heart disease and cerebrovascular disease)—was determined based on any of the following criteria: (1) a physician-confirmed diagnosis documented in the medical record; (2) self-report of a prior physician diagnosis. Trained research staff measured height and weight using calibrated electronic instruments (height: m; weight: kg; recorded to the nearest 0.01 units). Body mass index (BMI) was computed using the formula: weight (kg)/height² (m²), with values rounded to two decimal places. Participants were categorized according to Chinese BMI criteria: Underweight/normal weight: BMI < 24 kg/m^2^; Overweight: 24 ≤ BMI < 28 kg/m^2^; Obesity: BMI ≥ 28 kg/m^2^, Dual verification and quality control measures were implemented throughout the data collection process.

Following an overnight fast of >8 hours, peripheral venous blood (10 mL) was collected from all participants by trained nursing staff. The following biochemical parameters were quantified using an automated biochemical analyzer: Triglyceride (TG), Low-density lipoprotein cholesterol (LDL-C), HDL-C, Lipoprotein(a) [Lp(a)], Total cholesterol (TC), Glucose (Glu), Serum creatinine (Scr), SUA, Blood urea nitrogen (BUN), Total protein (TP), Albumin (Alb), Alkaline phosphatase (ALP), Alanine aminotransferase (ALT), Gamma-glutamyl transferase (GGT), Aspartate aminotransferase (AST).

### Statistical analysis

Normality assessment for continuous variables was performed using a combination of histograms, Q-Q plots, and Shapiro-Wilk tests. Continuous variables with normal distribution are reported using mean ± standard deviation, those with skewed distribution as median (interquartile range), and categorical variables as frequency counts and percentages. To evaluate baseline characteristic differences across UHR-subgroup groups, continuous variables were analyzed using ANOVA or Kruskal-Wallis tests as appropriate for distribution non normality, while categorical variables were assessed with the chi-square test. To ensure the robustness of regression analyses, potential outliers were systematically identified and evaluated. This was performed primarily through graphical inspection of standardized residual plots and leverage statistics. Multicollinearity was assessed using the variance inflation factor (VIF). Variables with a VIF > 5 were considered to exhibit severe multicollinearity and were excluded to ensure model stability. Multivariable linear regression was subsequently performed to examine the association of UHR with FR-BMD among the middle-aged and elderly cohort, with adjustment for all available covariates. Subgroup analyses by age, sex, and BMI were conducted, and interaction terms were incorporated to assess interaction effect. To investigate potential nonlinear associations of UHR with FR-BMD, generalized additive models (GAM) incorporating smoothing curves were applied. Threshold and saturation effect analyses identified inflection points in this association, followed by construction of piecewise linear regression models subgroup by these threshold values. Statistical computation was executed utilizing R (version 4.4.1) and EmpowerStats (version 4.2). Two-sided tests were used, and a *P*-value < 0.05 was considered statistically significant. Given the exploratory nature of the subgroup and nonlinear analyses, *P*-values are reported without correction for multiple comparisons.

## Results

### The basic characteristics of the study participants

The study cohort consisted of 4,958 participants averaging 60.77 ± 8.50 years, including 3,223 (65.0%) males and 1,735 (35.0%) females. All baseline characteristics demonstrated statistically significant differences (P < 0.05) when subgroup by UHR quartiles, with the exception of AST, total protein, and history of cerebrovascular disease. Individuals within the top UHR quartile (Q4) demonstrated a pronounced male predominance (86% *vs*. 14% females) and displayed significantly reduced levels (P<0.05) of age, ALP, LDL-C, HDL-C, TC and Lp(a) relative to the bottom quartile (Q1). Conversely, they showed significantly higher levels (P<0.05) of BMI, Alb, ALT, BUN, Scr, GGT, Glu, SUA, TG T-scores and FR-BMD. Additionally, Q4 participants had significantly higher prevalence rates of hypertension and diabetes but lower osteoporosis prevalence than Q1 counterparts. Detailed baseline characteristics are presented in **Table 1**.

**Table 1:** Baseline characteristics of different UHR groups^a^.

Level	Q1	Q2	Q3	Q4	*P*
n	1240	1240	1239	1239	
Sex, n (%)					<0.001
Male	437 (35.20)	754 (60.80)	966 (78.00)	1066 (86.00)	
Female	803 (64.80)	486 (39.20)	273 (22.00)	173 (14.00)	
Age (years)	61.28 ± 8.67	61.02 ± 8.30	60.88 ± 8.59	59.93 ± 8.36	<0.001
BMI, n (%)	23.82 ± 3.11	25.16 ± 3.04	26.13 ± 2.91	26.91 ± 2.88	<0.001
Albumin (g/L)	45.30 ± 2.78	45.51 ± 2.67	45.60 ± 2.71	45.68 ± 2.66	0.003
Alkaline phosphatase(U/L)	78.00(65.00,92.00)	76.50(63.75,91.00)	74.00(62.00,87.00)	73.00(61.00,87.00)	<0.001
Aspartate aminotransferase (U/L)	19.00(17.00,22.00)	19.00(16.00,22.00)	19.00(16.00,23.00)	19.00(16.00,23.00)	0.339
Alanine aminotransferase (U/L)	15.00(12.00,20.00)	16.00(13.00,21.00)	18.00(14.00,24.00)	19.00(15.00,26.00)	<0.001
Blood urea nitrogen(mmol/L)	4.60(3.90,5.40)	4.70(4.10,5.60)	4.80(4.10,5.70)	5.00(4.30,5.80)	<0.001
Total protein (g/L)	70.32 ± 4.05	70.61 ± 3.98	70.56 ± 3.99	70.68 ± 3.83	0.121
Serum creatinine (μmol/L)	59.55 ± 11.77	65.77 ± 12.30	70.75 ± 13.32	74.71 ± 13.52	<0.001
Gamma-glutamyltransferase(U/L)	16.00(12.00,21.25)	20.00(15.00,29.00)	23.00(16.00,33.50)	28.00(20.00,40.00)	<0.001
LDL-C(mmol/L)	3.12 ± 0.87	3.12 ± 0.90	3.04 ± 0.89	2.91 ± 0.86	<0.001
Glucose(mmol/L)	5.06(4.74,5.55)	5.18(4.81,5.84)	5.23(4.81,5.89)	5.30(4.89,5.93)	0.002
SUA (mg/dL)	3.78 ± 0.75	4.68 ± 0.77	5.35 ± 0.83	6.41 ± 1.10	<0.001
HDL-C (mg/dL)	64.48 ± 12.27	52.83 ± 8.68	46.21 ± 7.28	38.30 ± 6.74	<0.001
Total cholesterol (mmol/L)	5.13 ± 0.96	4.93 ± 1.00	4.78 ± 0.99	4.68 ± 0.98	<0.001
Triglyceride (mmol/L)	0.99(0.75,1.32)	1.21(0.90,1.65)	1.42(1.05,1.96)	1.86(1.33,2.59)	<0.001
Lipoprotein(a) (mg/L)	115.00(52.00,299.60)	104.20(47.50,239.43)	90.00(41.70,235.45)	76.30(36.70,180.60)	<0.001
Hypertension, n (%)					<0.001
No	766 (61.80)	730 (58.90)	715 (57.70)	653 (52.70)	
Yes	474 (38.20)	510 (41.10)	524 (42.30)	586 (47.30)	
Diabetes, n (%)					0.068
No	1101 (88.80)	1074 (86.60)	1065 (86.00)	1058 (85.4)	
Yes	139 (11.20)	166 (13.40)	174 (14.00)	181 (14.6)	
Cerebrovascular Diseases, n (%)					0.330
No	1225 (98.80)	1227 (99.00)	1216 (98.10)	1220 (98.50)	
Yes	15 (1.20)	13 (1.00)	23 (1.90)	19 (1.50)	
Coronary Heart Disease, n (%)					0.036
No	1202 (96.90)	1197(96.50)	1182 (95.40)	1176 (94.90)	
Yes	38 (3.10)	43 (3.50)	57 (4.60)	63 (5.10)	
T value	-1.23 ± 1.74	-1.09 ± 1.49	-0.95 ± 1.38	-0.86 ± 1.37	<0.001
BMD (g/cm2)	0.62 ± 0.12	0.66 ± 0.11	0.68 ± 0.10	0.70 ± 0.10	<0.001
Osteoporosis, n (%)					<0.001
No	955 (77.0)	1028 (82.9)	1087 (87.7)	1097(88.5)	
Yes	285 (23.0)	212(17.1)	152 (12.3)	142 (11.5)	

a BMI, body mass index; LDL-C, low-density lipoprotein cholesterol; HDL-C, high-density lipoprotein cholesterol; SUA, serum uric acid; BMD, bone mineral density.

### Association between UHR and BMD

**Figure 1:**
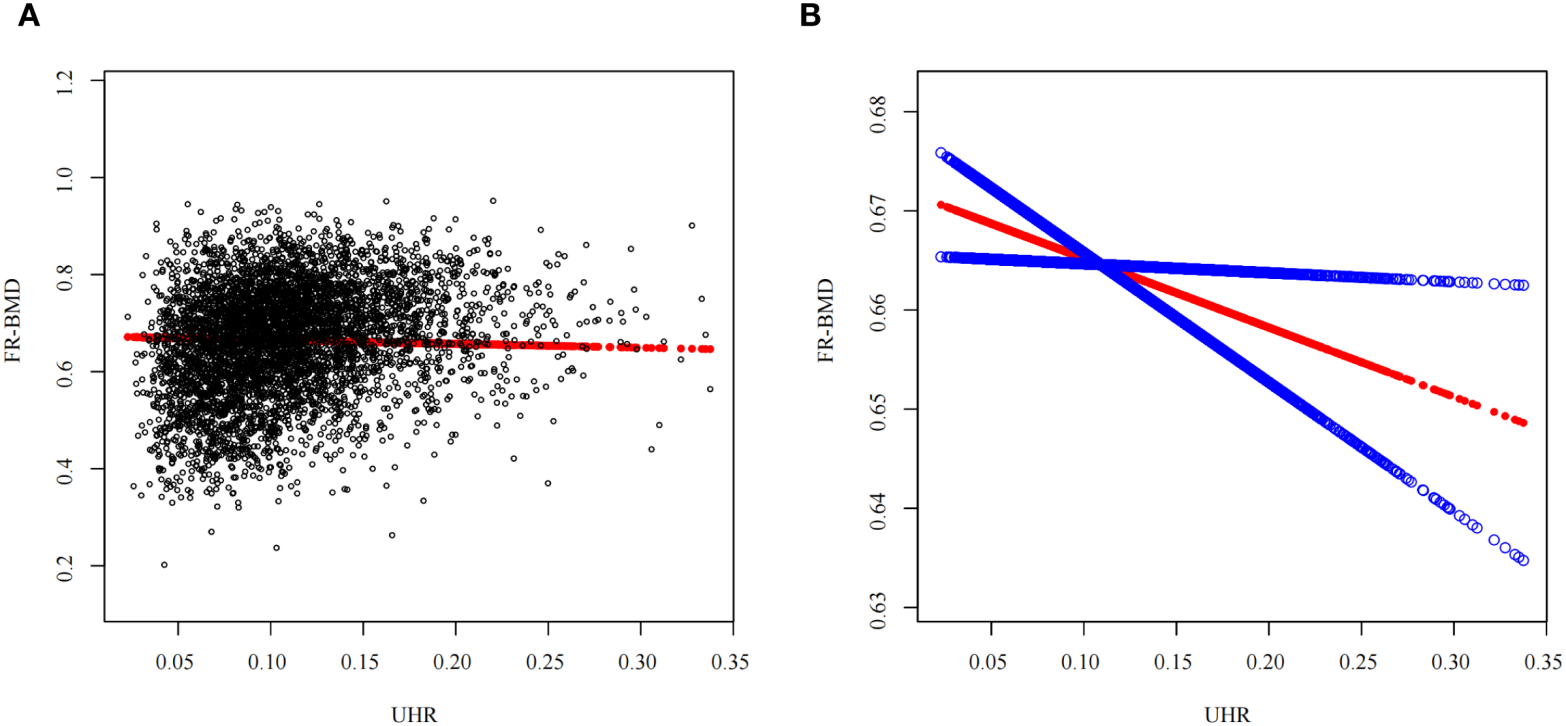
A non-linear association linking UHR to FR-BMD^a a^**(A)** Individual sample are represented by black circular markers in the plot; **(B)** The smoothed association between variables is illustrated by the solid red curve, with the surrounding blue band indicating the 95% confidence interval of the fitted relationship. Adjustments were made for all covariates.

Univariate linear regression was conducted using FR-BMD as the outcome variable. Independent variables included age, sex, BMI, Alb, ALP, AST, ALT, BUN, TP, Scr, GGT, LDL-C, Glu, SUA, LDL-C, TG, Lp(a), TC, coronary heart disease, diabetes, cerebrovascular disease, hypertension and osteoporosis. Results identified age, sex, BMI, Alb, ALP, ALT, BUN, TP, Scr, Lp(a), TC and hypertension as significant determinants of FR-BMD (*P*<0.05). Multivariable linear regression was subsequently performed using these statistically significant variables as covariates to assess the association of UHR with FR-BMD. In the crude analysis (Model 1, **Table 2**), UHR demonstrated a significant positive association with FR-BMD [*β* = 0.597, 95%CI(0.530~0.665), *P* < 0.001]. Given that the interquartile range (IQR) of UHR in our cohort is 0.058 (from 0.076 to 0.134), a 1-unit increase represents a change far exceeding the typical variation observed in this population. Therefore, the magnitude of the β-coefficient should be interpreted with this scale in mind. Following comprehensive adjustment for all covariates (Model 3, **Table 3**), a statistically significant inverse UHR-FR-BMD relationship was observed. Each 1-unit increase in UHR corresponded to an average decrease of 0.076g/cm² for FR-BMD[*β* = -0.076,95%CI(-0.138~-0.015), *P* = 0.015]. When the UHR was converted from a continuous variable to a categorical variable based on quartiles, linear regression analysis revealed that the highest UHR quartile (Q4) was associated with significantly lower FR-BMD compared to the lowest quartile (Q1) [*β* = -0.009,95%CI(-0.017~-0.001),*P* = 0.028]. A significant trend was observed across the quartiles (*P for trend* = 0.040), as detailed in **Table 3**. Furthermore, a smooth curve fitting and a generalized additive model were employed to assess the nonlinear association between UHR and FR-BMD. The analysis revealed a significant linear negative correlation between them (*P* < 0.05). **Table 3**, **Figure 1** presents the detailed results. Multicollinearity assessment using variance inflation factors (VIF) showed all values < 5, indicating no significant multicollinearity between UHR and the included covariates.

**Table 2:** Association of UHR with FR-BMD.

	Model 1 *β* (95% CI) *P* value	Model 2 *β*(95% CI) *P* value	Model 3 *β*(95% CI) *P* value	*Pi* value
UHR	0.597(0.530,0.665)<0.001	-0.043(-0.103,0.016)0.152	-0.076(-0.138,-0.015)0.015	
UHR quartile				
Q1	Reference	Reference	Reference	
Q2	0.035(0.026,0.043)<0.001	-0.004(-0.011,0.002)0.204	-0.005(-0.012,0.001)0.115	
Q3	0.062(0.053,0.070)<0.001	-0.004(-0.011,0.004)0.331	-0.006(-0.013,0.001)0.099	
Q4	0.076(0.067,0.084)<0.001	-0.006(-0.013,0.002)0.140	-0.009(-0.017,-0.001)0.025	
P for trend	<0.001	0.197	0.035	
Stratification by age^a^				<0.001
<60	0.491(0.414,0.568)<0.001	-0.044(-0.123,0.034)0.270	-0.062(-0.144,0.019)0.133	
≥60	0.632(0.520,0.745)<0.001	0.030(-0.062,0.121)0.527	0.014(-0.082,0.110)0.773	
Stratification by sex^b^				<0.001
Male	0.025(-0.042,0.092)0.458	-0.056(-0.125,0.014)0.117	-0.091(-0.163,-0.019)0.013	
Female	0.074(-0.054,0.203)0.254	0.169(0.060,0.279)0.002	0.153(0.040,0.265)0.008	
Stratification by BMI^c^				0.428
<24	0.731(0.583,0.879)<0.001	-0.024(-0.142,0.094)0.692	-0.068(-0.192,0.057)0.287	
24-28	0.496(0.394,0.599)<0.001	-0.074(-0.158,0.010)0.084	-0.093(-0.181,-0.005)0.039	
≥28	0.467(0.333,0.601)<0.001	0.036(-0.081,0.154)0.545	-0.017(-0.138,0.104)0.783	

Model 1: unadjusted; Model 2: adjusted for age, sex, and BMI; Model 3: fully adjusted. ^a^Age was not adjusted. ^b^sex was not adjusted. ^c^BMI was not adjusted. *P_i_*:The P-value of the interaction test.

**Table 3:** Sex differences in the relationship between UHR and forearm BMD in different age groups^a^.

Age group	Sex	*β* value	95%CI	*P-*value	*P_a_*-value
<60	Male	-0.096	(-0.183,-0.009)	0.031	0.097
	Female	0.058	(-0.105,0.220)	0.487	
≥60	Male	-0.140	(-0.240,-0.039)	0.006	<0.001
	Female	0.278	(0.126,0.430)	<0.001	

a Adjustments were made for all covariates except age and sex. *P_a_*-value: The statistical effect of sex differences within this age group.

**Figure 2:**
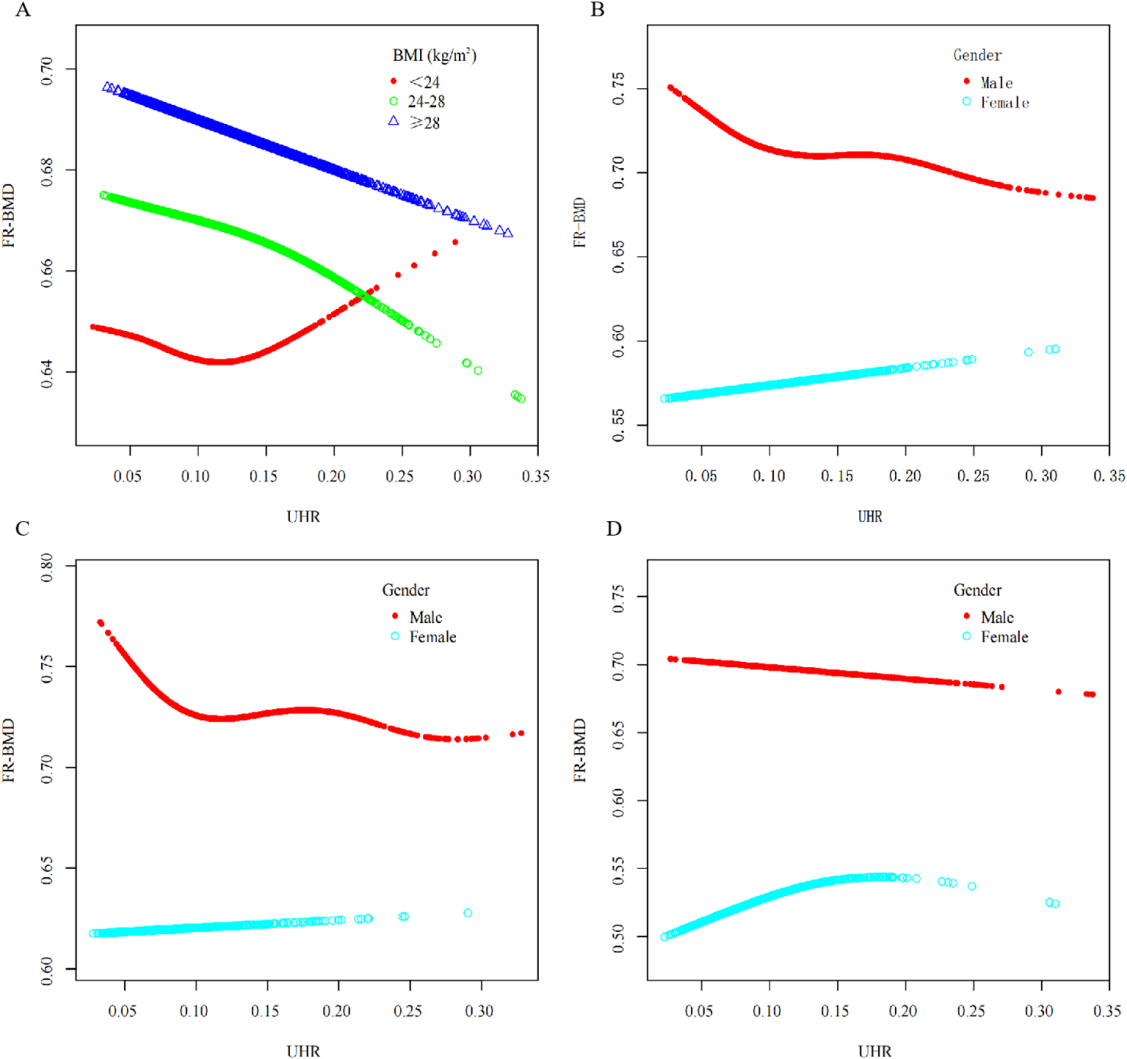
The non-linear UHR-FR-BMD association in subgroup analyses^a^. ^a^All covariates were adjusted for except those used for stratification. **(A)** stratified by BMI; **(B)** stratified by sex; **(C)** age<60 years old, stratified by sex; **(D)** age ≥ 60 years old, stratified by sex.

To explore potential variations in the association of UHR with FR-BMD across different population subgroups, we conducted a subgroup analysis by age, sex, and BMI. After adjustment for all covariates, the negative correlation was more pronounced in males [*β* = -0.091, 95%CI(-0.163~-0.019),*P* = 0.013] and overweight individuals [*β* = -0.093,95%CI(-0.181~-0.005), *P* = 0.039]. In contrast, a significant positive association was observed in female participants [*β* = 0.153,95%CI(0.040~0.265), *P* = 0.008]. No statistically significant associations were found in the remaining subgroups (P > 0.05). Detailed results are presented in **Table 3**, **Figure 2**. Interaction tests revealed a statistically significant age-by-sex interaction effect on the relationship between UHR and FR-BMD (*P for interaction*<0.05), indicating differential association patterns across age-sex strata. To ascertain the sex-based differences, we compared the relationship of UHR with FR-BMD between male and female participants within each age stratum. The results revealed a significant negative association between UHR and FR-BMD in male participants aged <60 years [*β* = -0.096, 95%CI(-0.183~-0.009), *P* = 0.031], whereas no significant association was observed in their female counterparts [*β* = 0.058, 95%CI(-0.105~0.220), *P* = 0.487]. However, a significant sex-specific interaction was found in the ≥60 years age group (*P_a_*<0.05). Specifically, the negative association remained significant in males [*β* = -0.140, 95%CI(-0.240~-0.039), *P* = 0.006]. Conversely, a significant positive association was identified in females, with a 1% increase in UHR corresponding to an average increase of 0.278 units in FR-BMD [*β* = 0.278, 95%CI(0.126~0.430), *P*<0.001]. These results are detailed in **Table 2**. The nonlinear trend is shown in **Figure 2**.

A U-shaped correlation of UHR with FR-BMD was exclusively identified in participants with BMI below 24 kg/m², reaching its inflection at 0.102. Piecewise linear regression modeling revealed that when UHR was below this threshold (UHR<0.102), each 1-unit increase in UHR corresponded to a significant 0.350g/cm² decrease in FR-BMD[β=-0.350,95%CI(-0.638~-0.062),*P* = 0.017]. Above the inflection point (UHR ≥ 0.102), this significant association was abolished. Complete results are presented in **Supplementary Table S1**. Among female participants aged ≥60 years, an inverted U-shaped curve characterized the relationship between UHR and FR-BMD, with an inflection point identified at 0.156. Piecewise linear regression modeling demonstrated that below this threshold (UHR<0.156), each unit increase in UHR corresponded to a progressive increase in FR-BMD[β=0.343, 95%CI(0.142~0.543), *P* < 0.001]. Beyond the inflection point (UHR ≥0.156), this significant association was no longer observed. Complete results are presented in **Supplementary Table S2**. In male participants aged <60 years, a nonlinear relationship was identified, characterized by a threshold at UHR = 0.072 (**Figure 2C**). The smoothed curve displayed an initial sharp decline in FR-BMD at very low UHR levels, which plateaued as UHR increased. The two-piecewise linear regression model determined the inflection point to be 0.072. The results indicated that when UHR was below 0.072, it showed a significant negative correlation with FR-BMD [*β* = -1.913, 95%CI(-3.420~-0.400), *P* = 0.013]. In contrast, no significant association was observed when UHR exceeded 0.072 [*β* = -0.023, 95%CI(-0.124~0.078),*P* = 0.650]. Detailed results are presented in **Supplementary Table S3**.

## Discussion

This research examined the relationship between UHR and FR-BMD in a sample of 4,958 adults aged 50 years or older. Multivariable linear regression analyses adjusted for confounders revealed a significant inverse association between UHR and FR-BMD. This relationship demonstrated significant modification through age-sex interaction effects. Specifically, we found a linear negative correlation in older men (≥ 60 years), a nonlinear pattern in middle-aged (< 60 years), and an intriguing inverted U-shaped relationship (with an inflection point at 0.156) in older women. In contrast, no significant association was observed in middle-aged women. A U-shaped relationship (reaching its inflection at 0.102) was observed in participants with BMI below 24kg/m². To the best of our understanding, no prior investigation has thoroughly evaluated the UHR and FR-BMD relationship, including its age-sex stratified variations and nonlinear trends. These findings offer novel insights into the metabolic-bone axis and advance osteoporosis risk assessment strategies.

The observed overall inverse association between UHR and FR-BMD may be interpreted through the integrated metabolic-inflammatory profile represented by this ratio. UHR encapsulates the balance between SUA and HDL-C, each with distinct roles in bone metabolism. SUA exhibits dual, concentration-dependent effects. At lower levels, it can function as an antioxidant, scavenging reactive oxygen species (ROS) to protect bone cells (11–13), and promote osteogenic signaling (14, 15). However, at elevated concentrations, it may exert pro-oxidant and pro-inflammatory effects, including the stimulation of osteoclastogenic cytokines, thereby promoting bone resorption (15, 16). Conversely, HDL-C exerts anti-inflammatory and antioxidant effects, and its higher levels are generally associated with better BMD—a benefit attributed to its role in mitigating osteoclastogenesis induced by pro-inflammatory cytokines and reducing oxidative stress in bone (17–19). Therefore, a higher UHR signifies a relative excess of pro-inflammatory/pro-oxidant SUA alongside a deficiency of protective HDL-C. This imbalance likely promotes a bone microenvironment characterized by heightened oxidative stress and chronic low-grade inflammation, which can collectively enhance osteoclast activity, impair osteoblast function, and shift bone remodeling towards net resorption. This framework provides a plausible biological basis for the inverse UHR-FR-BMD association and underscores UHR’s potential as an integrative marker of systemic metabolic bone risk. Furthermore, it helps contextualize the distinct, subgroup-dependent associations we identified, suggesting that the net impact of this metabolic balance is modulated by factors such as age, sex, and BMI.

The relationship between UHR and BMD reported in the literature is heterogeneous, underscoring the importance of population context. Our finding of an inverse UHR-BMD association in a general middle-aged and elderly Chinese cohort contrasts with several previous reports. For instance, studies on non-diabetic middle-aged and elderly adults in China have reported a positive correlation between UHR and BMD at major weight-bearing sites (20), with one study further indicating that elevated UHR levels were associated with a reduced incidence of osteoporosis (21). Similarly, analyses of the U.S. NHANES population revealed a positive correlation between UHR and femoral neck BMD; notably, this association was significant only in individuals aged ≥65 years, but not in those aged 50–64 years (22). Conversely, investigations in specific patient groups (type 2 diabetes (23, 24) or spinal degeneration (25) reported that lower UHR was a risk factor for reduced BMD. Several factors may explain these discrepant findings. First, differences in population characteristics are crucial: our study focused on a Chinese middle-aged and elderly health-examination cohort, whereas prior studies largely involved general U.S. populations or specific disease-based groups such as type 2 diabetes patients. Variations in genetic background, lifestyle, and comorbid conditions may fundamentally alter the biological effects of uric acid and HDL-C on bone metabolism. Second, the site of BMD measurement must be considered: most studies reporting positive associations measured BMD at weight-bearing sites (e.g., lumbar spine, hip), where bone metabolism is influenced substantially by biomechanical loading. In contrast, our study primarily assessed forearm BMD—a non-weight-bearing site that may be more sensitive to systemic metabolic disturbances reflected by the UHR.

Most importantly, our study identified significant interaction effect by age and sex, providing a key framework for reconciling earlier inconsistencies. For instance, while NHANES research suggested age-related heterogeneity in this association, our findings further demonstrate that among Chinese adults aged ≥50 years, the UHR-BMD relationship not only differs in strength but can also reverse direction (e.g., positive in older women) or exhibit nonlinear patterns across age-sex subgroups. This indicates that the oxidative-inflammatory balance represented by a high UHR is modulated by demographic and physiological context. Thus, our results suggest that the UHR-BMD association is not universal but highly dependent on key effect modifiers, highlighting the importance of subgroup analysis in future research and clinical interpretation.

In our Chinese health examination cohort, after comprehensive adjustment for confounders (including age, sex, BMI, Alb, ALP, ALT, BUN, TP, Scr, Lp(a), TC and hypertension), elevated UHR demonstrated a significant inverse association with FR-BMD. This suggests that the metabolic imbalance profile characterized by high SUA and low HDL-C – reflected by increased UHR – may adversely impact bone density through pro-oxidative effects and pro-inflammatory cytokine release. These mechanisms exacerbate oxidative stress and chronic inflammation, ultimately disrupting the bone formation-resorption equilibrium. Significant age-by-sex interaction (*P for interaction*<0.05) indicates the inverse UHR-BMD relationship is context-dependent rather than universally applicable, being substantially modified by age-sex strata. Notably, in fully adjusted models, a positive correlation of UHR with FR-BMD was observed among female participants aged ≥ 60 years, with a further inverted U-shaped relationship identified (inflection point at UHR = 0.156). This reversal from the overall inverse trend warrants a focused mechanistic interpretation, likely centered on the profound endocrinological shift of menopause. The abrupt decline in estrogen following menopause creates a unique metabolic and inflammatory milieu that may fundamentally alter the biological interpretation of UHR. Estrogen deficiency upregulates pro-inflammatory cytokines (e.g., TNF-α, IL-1, IL-6), accelerating bone resorption via the RANKL/RANK/OPG pathway (26, 27); impairs antioxidant defenses, leading to elevated oxidative stress in bone cells (28); and directly diminishes osteoblast activity (29–31). In this context, the components of UHR may play altered roles. Below the inflection point (UHR < 0.156), the observed positive association may be explained by a shift in the balance of UHR’s components: In a state of heightened postmenopausal oxidative stress, moderately elevated SUA may exert a more pronounced antioxidant effect, scavenging reactive ROS and partially counteracting the oxidative damage to osteoblasts and osteocytes (32, 33). This protective role might temporarily outweigh its pro-inflammatory potential. Concurrently, a relatively higher HDL-C level (contributing to a lower UHR) would maintain its anti-inflammatory and antioxidant vascular-protective functions, potentially supporting bone perfusion and a healthier bone microenvironment (34, 35). Thus, in older women, a moderately elevated UHR within this range might paradoxically signal a more favorable oxidative-inflammatory balance for bone. Above the inflection point (UHR ≥ 0.156), the relationship plateaus and reverses, suggesting a threshold effect. Excessively high SUA levels may transition to exhibit predominant pro-oxidant and pro-inflammatory properties, promoting cytokine production and ROS generation (15, 36).

Simultaneously, a critically low HDL-C level would signify a loss of its protective functions. Beyond this threshold, the detrimental metabolic syndrome-like profile (high SUA, low HDL-C) likely overwhelms any residual antioxidant benefit of SUA, realigning with the adverse association seen in other subgroups. Additionally, very high SUA levels may be associated with subclinical renal impairment, affecting its clearance and further complicating its relationship with bone (37). This context-dependent duality of UHR—where its effect is modulated by the underlying endocrine and inflammatory status—explains not only the reversal in older women but also strengthens the interpretation of the negative association in men and middle-aged women, whose hormonal environments favor different metabolic set-points. These mechanistic speculations, while requiring direct validation, highlight UHR not as a univariate risk factor but as a dynamic integrator of metabolic health whose clinical interpretation is inseparable from the patient’s age and sex.

Subgroup analysis by age and sex revealed a significant inverse association between UHR and BMD in the middle-aged and elderly male population. This may be attributed to distinct physiological contexts: in male s, persistently low yet stable estrogen levels minimally influence bone metabolism, which is predominantly regulated by androgens and complex multifactorial mechanisms, potentially reducing UHR sensitivity (38, 39). In premenopausal women (<60 years), the absence of sharp estrogen decline likely preserves antioxidant and anti-inflammatory reserves, enhancing resilience to UHR fluctuations. These findings are consistent with established evidence identifying postmenopausal women as the most vulnerable population for bone metabolic dysregulation (40).

Existing evidence indicates that elevated BMI increases skeletal loading, which stimulates osteoblast proliferation/differentiation while suppressing osteoclast activity, thereby promoting bone formation and benefiting skeletal health (41). Consistent with prior research, our study identified low BMI as a risk factor for reduced BMD, with higher BMI values correlating with increased BMD. Notably, a U-shaped correlation linking UHR to FR-BMD was observed in participants with BMI < 24 kg/m². This may reflect diminished mechanical loading effects in normal/low-BMI individuals, weakening the protective skeletal adaptation to weight-bearing stimuli and thereby their bone metabolism becomes more vulnerable to metabolic fluctuations and modulators including age, hormonal status, and lifestyle factors (42). The precise mechanisms underlying these associations remain to be elucidated and warrant further investigation.

This study represents the first large-scale investigation to systematically examine the association of UHR with FR-BMD in adults aged 50 years and older, with comprehensive analysis of age-sex stratification patterns and nonlinear associations. Our findings suggest that UHR could be a novel integrated biomarker that may shows a closer association with bone metabolic dysregulation than individual parameters. Crucially, assessment of UHR’s impact on BMD should consider careful consideration of age, sex, and BMI stratification. Specifically, maintaining moderately elevated UHR levels (UHR<0.156) may confer skeletal benefits in women aged ≥ 60 years. Similarly, maintaining moderately elevated UHR levels may confer a protective effect against osteoporosis in middle-aged and elderly men, as well as in individuals with a BMI of less than 24 kg/m². These insights preliminarily highlight the potential of UHR as a stratification tool in osteoporosis risk assessment; however, its utility in targeted intervention strategies tailored to specific population subgroups needs to be established in future studies.

Several limitations should be considered in this study: First, owing to its cross-sectional nature, this study is unable to infer causal relationships linking UHR to FR-BMD. Second, selection bias may exist as participants were recruited exclusively from health examination populations. Third, BMD was measured solely at the forearm, a non-weight-bearing site. While this may enhance sensitivity to systemic metabolic disturbances, it limits direct comparability with studies using hip or spine BMD and may not fully represent osteoporosis risk at clinically relevant fracture sites. Fourth, due to the constraints of the retrospective health examination data source, key covariates including medication history, lifestyle factors, and laboratory parameters (e.g., serum calcium and 25-hydroxyvitamin D) were not assessed and represent potential unmeasured confounders. Finally, the study involved multiple subgroup comparisons and nonlinearity tests without statistical adjustment for multiple testing(e.g., Bonferroni). This exploratory approach increases the risk of false-positive findings, and therefore the identified subgroup-specific associations and nonlinear patterns require validation in independent cohorts.

## Conclusion

Our study demonstrates that the relationship between UHR and FR-BMD is significantly influenced by age and sex in middle-aged and elderly populations. Specifically, FR-BMD showed a declining trend with increasing UHR in middle-aged and elderly men. In contrast, an inverted U-shaped association was observed in women ≥ 60 years, and a U-shaped relationship was found in individuals with a BMI<24 kg/m^2^. These exploratory findings suggest that UHR may serve as a novel metabolic biomarker for skeletal health assessment, with effects that vary across subgroups. This study provides new epidemiological evidence for the association between UHR and BMD, which could support further exploration of its utility in osteoporosis risk assessment. Future large-scale prospective cohort studies are needed to validate the UHR-BMD relationship. Mechanistic investigations into UHR’s role in bone metabolism regulation are also warranted.

## Clinical perspectives

Background as to why the study was undertaken: The UHR has emerged as a novel biomarker associated with various metabolic diseases, but its relationship with BMD, particularly in the context of age and sex differences, remains unclear. This study was undertaken to definitively assess the association between UHR and forearm BMD in a large middle-aged and elderly Chinese cohort and to investigate the potential modifying effects of key demographic factors.

A brief summary of the results: This study identified a significant inverse association between UHR and forearm BMD after adjusting for confounders, which was markedly modified by age and sex. Notably, a positive association was found in females aged ≥60 years, while a negative correlation was observed in males and overweight individuals. Furthermore, nonlinear U-shaped and inverted U-shaped relationships were detected in non-overweight individuals and older females, respectively.

The potential significance of the results to human health and disease: These findings position UHR as a potential novel, cost-effective integrative biomarker for assessing bone health and osteoporosis risk. The results underscore the importance of demographic-stratified approaches, suggesting that maintaining UHR within a specific moderate range could be a beneficial strategy for osteoporosis prevention in postmenopausal women and non-overweight individuals, thereby informing more personalized public health and clinical interventions.

## Data Availability

The raw data supporting the conclusions of this article will be made available by the authors, without undue reservation.

## Supplementary material

Supplementary Table 1 Threshold effect analysis of UHR on FR-BMD in BMI<24kg/m^2^ using the two-piecewise linear regression model^a^.

Supplementary Table 2 Threshold effect analysis of UHR on FR-BMD in female aged 60 or above using the two-piecewise linear regression model^a^.

Supplementary Table 3 Threshold effect analysis of UHR on FR-BMD in males aged <60 years using the two-piecewise linear regression model^a^.
