# Supplementary material for: Association between the serum uric acid to HDL cholesterol ratio and forearm bone mineral density in middle-aged and older adults
Source: Front Endocrinol (Lausanne). 2026 Feb 11;17:1710027. doi: 10.3389/fendo.2026.1710027 (PMC12932171; doi:10.3389/fendo.2026.1710027)
Supplement: Supplementary Table 1 — Threshold effect analysis of UHR on FR-BMD in BMI<24kg/m2 using the two-piecewise linear regression modela. [file Table1.docx]

**Supplementary Material for Association between the serum uric acid to HDL cholesterol ratio and forearm bone mineral density in middle-aged and older adults**

**Supplementary Table S 1** Threshold effect analysis of UHR on FR-BMD in BMI＜24kg/m^2^ using the two-piecewise linear regression model^a^

| Forearm bone mineral density | Adjusted *β (95% CI) P* value |
| --- | --- |
| BMI＜24kg/m^2^ |  |
| Fitting by the standard linear model | -0.076(-0.204,0.052) 0.244 |
| Fitting by the two-piecewise linear model | |
| Inflection point | 0.102 |
| UHR<0.102 | -0.350(-0.638, -0.062) 0.017 |
| UHR>0.102 | 0.139(-0.104, 0.382) 0.262 |
| Log likelihood ratio | 0.009 |

^a^Adjustments were made for all covariates

**Supplementary Table S 2** Threshold effect analysis of UHR on FR-BMD in female aged 60 or above using the two-piecewise linear regression model^a^

| Forearm bone mineral density | Adjusted *β (95% CI) P* value |
| --- | --- |
| Age≥60, Female |  |
| Fitting by the standard linear model | 0.245(0.084, 0.405) 0.003 |
| Fitting by the two-piecewise linear model | |
| Inflection point | 0.156 |
| UHR<0.156 | 0.343(0.142,0.543)＜0.001 |
| UHR>0.156 | -0.274(-0.949,0.401) 0.427 |
| Log likelihood ratio | 0.048 |

^a^Adjustments were made for all covariates except age and sex.

**Supplementary Table S 3** Threshold effect analysis of UHR on FR-BMD in males aged <60 years using the two-piecewise linear regression model^a^

| Forearm bone mineral density | Adjusted *β (95% CI) P* value |
| --- | --- |
| Age ≥ 60, male |  |
| Fitting by the standard linear model | -0.077(-0.172,0.017) 0.108 |
| Fitting by the two-piecewise linear model | |
| Inflection point | 0.072 |
| UHR<0.072 | -1.913(-3.420,-0.400) 0.013 |
| UHR>0.072 | -0.023(-0.124,0.078) 0.650 |
| Log likelihood ratio | ＜0.001 |

^a^Adjustments were made for all covariates except age and sex.
